# The tumor-associated antigen RHAMM (HMMR/CD168) is expressed by monocyte-derived dendritic cells and presented to T cells

**DOI:** 10.18632/oncotarget.12170

**Published:** 2016-09-21

**Authors:** Yannick Willemen, Johan M.J. Van den Bergh, Sarah M. Bonte, Sébastien Anguille, Carlo Heirman, Barbara M.H. Stein, Herman Goossens, Tessa Kerre, Kris Thielemans, Marc Peeters, Viggo F.I. Van Tendeloo, Evelien L.J. Smits, Zwi N. Berneman

**Affiliations:** ^1^ Laboratory of Experimental Hematology, Vaccine & Infectious Disease Institute, University of Antwerp, Antwerp, Belgium; ^2^ Department of Hematology and Clinical Chemistry, Microbiology and Immunology, Ghent University, Ghent, Belgium; ^3^ Laboratory of Molecular and Cellular Therapy, Department of Immunology-Physiology, Vrije Universiteit Brussel, Brussels, Belgium; ^4^ Laboratory of Medical Microbiology, Vaccine & Infectious Disease Institute, University of Antwerp, Antwerp, Belgium; ^5^ Center for Oncological Research, University of Antwerp, Antwerp, Belgium

**Keywords:** electroporation, hyaluronan-mediated motility receptor, immunotherapy, messenger RNA, vaccination

## Abstract

We formerly demonstrated that vaccination with Wilms’ tumor 1 (WT1)-loaded autologous monocyte-derived dendritic cells (mo-DCs) can be a well-tolerated effective treatment in acute myeloid leukemia (AML) patients. Here, we investigated whether we could introduce the receptor for hyaluronic acid-mediated motility (RHAMM/HMMR/CD168), another clinically relevant tumor-associated antigen, into these mo-DCs through mRNA electroporation and elicit RHAMM-specific immune responses. While *RHAMM* mRNA electroporation significantly increased RHAMM protein expression by mo-DCs, our data indicate that classical mo-DCs already express and present RHAMM at sufficient levels to activate RHAMM-specific T cells, regardless of electroporation. Moreover, we found that RHAMM-specific T cells are present at vaccination sites in AML patients. Our findings implicate that we and others who are using classical mo-DCs for cancer immunotherapy are already vaccinating against RHAMM.

## INTRODUCTION

Despite major advances over the past decades, the prognosis of acute myeloid leukemia (AML) remains poor due to frequent relapse, which is related to minimal residual disease following initial treatment [1]. This holds particularly true for the large group of older patients with comorbidity deemed unfit to undergo intensive treatment and for whom median survival is less than one year. Immunotherapy, including dendritic cell (DC) vaccination, might offer an alternative. We demonstrated in a phase I/II clinical trial that autologous dendritic cells (DCs) loaded with the tumor-associated antigen (TAA) Wilms’ tumor 1 (WT1) by mRNA electroporation can induce clinical responses in AML patients with very limited side effects [2]. Because of the favorable toxicity profile and promising results in early phase clinical trials by ourselves and others, DC vaccination is widely under investigation in the treatment of various solid and hematological malignancies [2–4]. Simultaneously, research efforts are directed towards new strategies to improve the efficacy of DC vaccination, as objective and durable clinical responses are currently limited to a small minority of cancer patients [3–5].

Much interest goes to boosting the immunostimulatory properties of existing clinical DC vaccination protocols in order to improve their efficacy. It has become increasingly evident that the clinical success of DC vaccination – and immunotherapy in general – is hampered by cancer cells escaping immune control by passively and actively evading and suppressing immunity [6, 7]. The ideal DC vaccine should therefore contain potent immunostimulatory DCs that can activate a broad repertoire of tumor cell-specific T cells through presentation of different TAAs.

Correspondingly, adding another TAA, next to WT1, to our clinically used DCs would allow for a multi-antigen vaccine that may help withstand cancer immune escape in a more efficient manner. The receptor for hyaluronic acid-mediated motility (RHAMM/HMMR/CD168) is considered an interesting target for cancer immunotherapy, because it is a TAA expressed by a broad variety of hematological malignancies and solid tumors which is linked to tumor progression and has prognostic relevance [8–16]. In addition, murine bone marrow-derived DCs transfected with *RHAMM* mRNA were shown to significantly improve survival in a mouse glioma model [17]. Even if RHAMM was not overexpressed in leukemic cells with a stem cell immunophenotype, [18] RHAMM-specific T cells were able to control tumor growth in human xenograft solid tumor and disseminated AML mouse models [19]. Most importantly, clinical trials with RHAMM peptide vaccination have already demonstrated immunological and clinical responses in patients with various hematological malignancies, including AML, chronic lymphocytic leukemia, multiple myeloma and myelodysplastic syndrome [20–22].

In the present work, we examined whether RHAMM could be introduced into the human monocyte-derived (mo-)DCs we use in our cancer vaccine clinical trials through mRNA electroporation, and whether these DCs then present RHAMM and activate RHAMM-specific T cells.

## RESULTS

### *RHAMM* mRNA electroporation increases RHAMM protein expression by mo-DCs

**Figure 1:**
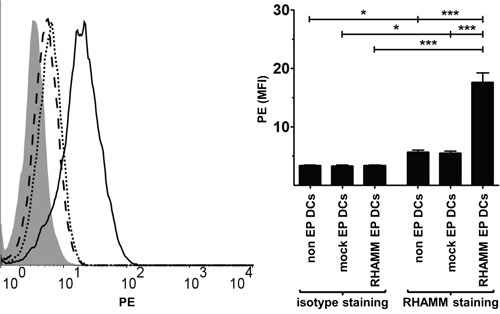
RHAMM protein expression in DCs Four hours after electroporation, non EP DCs, mock EP DCs and RHAMM EP DCs were stained with LIVE/DEAD® Fixable Red Stain prior to two-step intracellular staining with RHAMM or isotype control mouse IgG1 antibody and rat anti-mouse IgG1-PE. Samples were acquired on a FACScan flow cytometer. The histogram overlay shows PE staining levels of isotype stained RHAMM EP DCs (grey filled area) or RHAMM stained non EP DCs (dotted black line), mock EP DCs (dashed black line) and RHAMM EP DCs (full black line) from one representative donor. PE staining is further depicted as mean fluorescence intensity (+ SD) of viable (LIVE/DEAD^−^) DCs from 3 independent donors; *P < 0.05, ***P < 0.001, one-way ANOVA with Bonferroni posthoc test. MFI, mean fluorescence intensity.

We first tested whether DCs express RHAMM following mRNA electroporation by examining RHAMM protein levels in non EP DCs, mock EP DCs and RHAMM EP DCs using intracellular staining. RHAMM EP DCs clearly expressed the RHAMM protein following electroporation, as mean fluorescence intensity (MFI) of samples stained for RHAMM far exceeded that of the respective isotype stained control samples (MFI 17.7 and 3.5, respectively; *p* < 0.001; n = 3; Figure 1). These RHAMM protein levels in RHAMM EP DCs were significantly higher than those in non EP DCs and mock EP DCs (MFI 5.7 and 5.5, respectively; *p* < 0.001; n = 3; Figure 1). Interestingly, MFI of non EP DCs and mock EP DCs stained for RHAMM was higher than that of their respective isotype stained control samples (MFI 3.4 and 3.4, respectively; *p* < 0.05; n = 3; Figure 1). These data show that *RHAMM* mRNA electroporation of DCs leads to increased RHAMM protein expression, but also suggest that RHAMM is already expressed by mo-DCs irrespective of electroporation.

### *RHAMM* mRNA is expressed by mo-DCs

**Figure 2:**
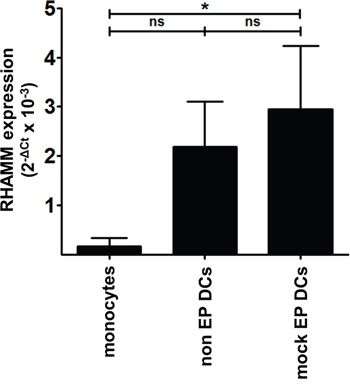
Native *RHAMM* mRNA expression levels in monocytes and mo-DCs Total cDNA from monocytes, non EP DCs and mock EP DCs served as template to determine *RHAMM* mRNA expression levels in these cells by qPCR. Results were analyzed using the ΔCt method and normalized to the mean of GAPDH and YWHAZ expression. Data are depicted as mean 2^−ΔCt^ values (+ SD) from 3 independent donors; ns not significant, *P < 0.05, one-way ANOVA with Bonferroni posthoc test.

To verify whether RHAMM is expressed by mo-DCs, we quantified *RHAMM* mRNA levels in monocytes, non EP DCs and mock EP DCs by quantitative real-time polymerase chain reaction (qPCR). Freshly isolated monocytes displayed low background *RHAMM* mRNA expression when normalized for two household genes (mean [2^−ΔCt^ x 10^−3^] 0.2; n = 3; Figure 2). Conversely, non EP DCs and mock EP DCs expressed detectable levels of *RHAMM* mRNA (mean [2^−ΔCt^ x 10^−3^] 2.2 and 2.9, respectively; n = 3; Figure 2). In addition to the evidence on protein level, these results confirm on the mRNA level that mo-DCs express RHAMM.

### Mo-DCs present RHAMM and activate RHAMM-specific cytotoxic T cells regardless of *RHAMM* mRNA electroporation

**Figure 3:**
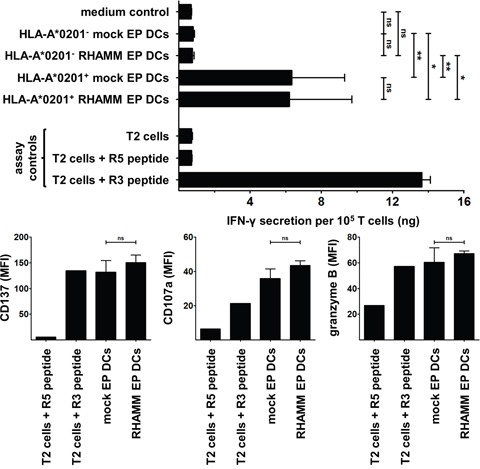
Stimulation of RHAMM R3-specific T cells by DCs The RHAMM R3-specific cytotoxic CD8^+^ T cell clone was stimulated for 24 hours without DCs (medium control) or with mock EP DCs or RHAMM EP DCs from HLA-A*0201^−^ (n = 5) or A*0201^+^ (n = 3) donors. T cells stimulated with T2 cells ± R3 or R5 peptide served as negative and positive controls. IFN-γ was quantified in culture supernatants using ELISA. Data are depicted as mean (+ SD); ns not significant, *P < 0.05, **P < 0.01, one-way ANOVA with Bonferroni posthoc test. CD137, CD107a and granzyme B expression by CD8^+^ T cells was determined by flow cytometry following 24-hour stimulation with mock EP DCs or RHAMM EP DCs from HLA-A*0201^+^ donors (n = 2). T cells stimulated with T2 cells + R3 or R5 peptide served as positive and negative controls, respectively. Data are depicted as mean fluorescence intensity (+SD); ns not significant, unpaired *t* test.

After establishing that mo-DCs express RHAMM, we sought to determine whether they can also activate RHAMM-specific CD8^+^ cytotoxic T cells through presentation of RHAMM epitopes in a human leukocyte antigen (HLA)-dependent manner. Mock EP DCs and RHAMM EP DCs from HLA-A*0201^−^ or A*0201^+^ donors were evaluated for their capacity to induce IFN-γ secretion or CD137, CD107a and granzyme B expression by the RHAMM R3-specific CD8^+^ cytotoxic T cell clone. T cells stimulated with mock EP DCs or RHAMM EP DCs from HLA-A*0201^−^ donors did not secrete more IFN-γ than T cells cultured alone (medium control; Figure 3). In contrast, culture with mock EP DCs or RHAMM EP DCs from HLA-A*0201^+^ donors significantly increased IFN-γ secretion by T cells (Figure 3). Despite notable differences in RHAMM protein expression, stimulation with mock EP DCs or RHAMM EP DCs from HLA-A*0201^+^ donors resulted in similar levels of IFN-γ secretion (mean 6.4 and 6.2 ng/10^5^ T cells, respectively; n = 3; Figure 3). Likewise, culture with mock EP DCs or RHAMM EP DCs from HLA-A*0201^+^ donors induced virtually equal levels of CD137, CD107a and granzyme B expression by T cells (Figure 3). These data indicate that mo-DCs present RHAMM and activate cytotoxic CD8^+^ T cells specific for this TAA in an HLA-dependent manner, and that this activation is not enhanced by increasing RHAMM protein expression via mRNA electroporation.

### Clinical vaccine WT1-loaded mo-DCs from AML patients express and present RHAMM

**Figure 4:**
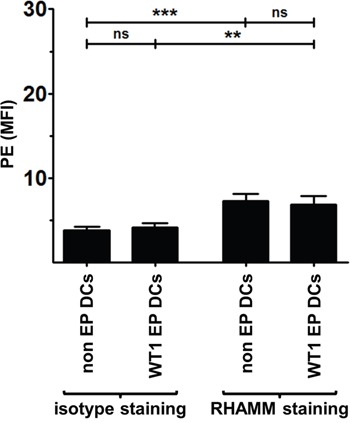
RHAMM protein expression in AML patient DCs Two hours after thawing, non EP DCs and WT1 EP DCs were stained with LIVE/DEAD® Fixable Red Stain prior to two-step intracellular staining with RHAMM or isotype control mouse IgG1 antibody and rat anti-mouse IgG1-PE. Samples were acquired on a FACScan flow cytometer. Data are presented as mean (+ SD) of viable (LIVE/DEAD^−^) DCs from 4 independent donors; ns not significant, **P < 0.01, ***P < 0.001, one-way ANOVA with Bonferroni posthoc test. MFI, mean fluorescence intensity.

**Figure 5:**
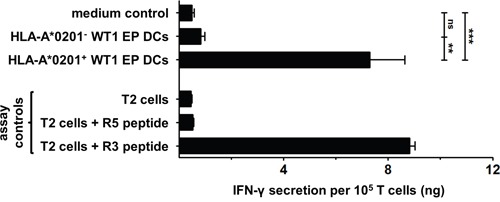
Stimulation of RHAMM R3-specific T cells by AML patient DCs The RHAMM R3-specific cytotoxic CD8^+^ T cell clone was stimulated for 24 hours without DCs (medium control) or with WT1 EP DCs from HLA-A*0201^−^ (n = 2) or A*0201^+^ (n = 3) donors. T cells stimulated with T2 cells ± R3 or R5 peptide served as intra-assay controls to validate antigen specificity. IFN-γ was quantified in culture supernatants using ELISA. Data are depicted as mean (+ SD); ns not significant, **P < 0.01, ***P < 0.001, one-way ANOVA with Bonferroni posthoc test.

We investigated RHAMM expression and presentation by thawed AML patients’ WT1 EP DCs used in our clinical vaccination trials. Similar to mo-DCs from healthy donors, AML patient WT1 EP DCs showed RHAMM protein expression levels which were comparable to that of non EP DCs from the same respective patients and exceeded isotype control background staining (n = 4; Figure 4). Moreover, we found that WT1 EP DCs from HLA-A*0201^+^ AML patient donors strongly stimulated IFN-γ secretion by the RHAMM R3-specific CD8^+^ cytotoxic T cell clone whereas those from HLA-A*0201^−^ AML patient donors did not (mean 7.3 and 0.8 ng/10^5^ T cells, respectively; *p* < 0.01; n = 3 and 2, respectively; Figure 5). These results show that the WT1-loaded mo-DCs used as autologous vaccines in AML patients also express the TAA RHAMM at sufficient levels to potently activate RHAMM-specific cytotoxic CD8^+^ T cells in an HLA-dependent manner.

### RHAMM-specific T cells are present in vaccination sites in AML patients

**Figure 6:**
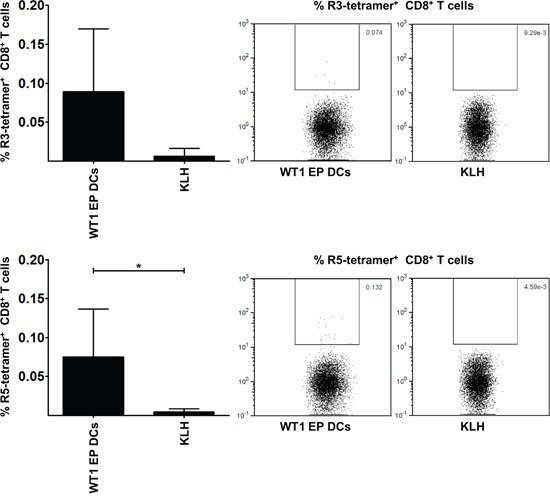
RHAMM-specific CD8+ T cells in vaccinated HLA-A*0201+ AML patients The graphs on the left show the percentage of RHAMM R3 and R5-specific cells within the CD8^+^ T cell population infiltrating the injection sites of WT1 EP DCs (i.e., the vaccine; n = 6) or KLH alone (n = 3). Samples were measured by flow cytometry following staining with R3 or R5 pHLA-A*0201 tetramers. Data are depicted as mean (+ SD); *P < 0.05, two-tailed Mann Whitney *U* test. On the right, dot plots show R3 or R5 pHLA-A*0201 tetramer staining results of CD8^+^ T cells isolated from WT1 EP DC or KLH injection site skin biopsies.

Finally, we examined *in vivo* T cell responses in HLA-A*0201^+^ AML patients vaccinated with WT1 EP DCs for the presence of RHAMM-specific T cells. The proportion of RHAMM R3- and R5-specific cells within the CD8^+^ T cell population infiltrating the injection sites of the vaccine (i.e. WT1 EP DCs) or keyhole limpet hemocyanin (KLH) alone, in the context of a delayed type hypersensitivity (DTH) skin test, was measured by flow cytometry following staining with R3 or R5 peptide-loaded (p)HLA-A*0201 tetramers. The percentages of RHAMM R3- and R5-specific T cells within the CD8^+^ T cell population isolated from the vaccine injection site (mean 0.089 and 0.075%, respectively; n = 6; Figure 6) were higher than those in the population isolated from the KLH injection site (mean 0.006 and 0.004%, respectively; n = 3; Figure 6). These differences were statistically significant for the R5 epitope and demonstrate that RHAMM-specific T cells are found at the injection sites of AML patients vaccinated with mo-DCs that were not specifically loaded with RHAMM mRNA.

## DISCUSSION

Clinical trials by our group and others have demonstrated that DC vaccination can induce objective clinical responses in cancer patients without significant toxicity, but at the same time highlighted that new strategies are needed to improve the efficacy of DC vaccination [2–5]. RHAMM is considered a promising and clinically relevant TAA, with studies showing that RHAMM peptide vaccination and RHAMM-specific immune responses can yield clinical effects [19–21]. We hypothesized that introducing RHAMM into our clinically used DC vaccine through mRNA electroporation might enhance efficacy by broadening the scope of anti-TAA immunity.

We first electroporated human mo-DCs with *RHAMM* mRNA and showed that this approach resulted in the expression of increased levels of RHAMM protein in mo-DCs. Interestingly, mo-DCs that were not electroporated or were electroporated without *RHAMM* mRNA also expressed RHAMM, albeit at lower levels. Natural *RHAMM* mRNA expression in these cells was further confirmed by qPCR. Other studies already reported that RHAMM expression is not restricted to malignant cells but is also found at varying levels in different normal cells and tissues, including testis and spermatocytes, placenta, thymus, the eye, parts of the gastro-intestinal mucosa, normal proliferating CD34^+^ cells and activated T cells [8, 15, 16, 18, 23]. Yet, this is the first report describing RHAMM expression by classical mo-DCs. This is most probably an effect resulting from cytokine stimulation and differentiation during *in vitro* culture, considering the virtual absence in monocytes and upregulation of RHAMM expression in other normal activated or dividing cells [18, 24–26].

We further showed that mock EP DCs induced similar levels of IFN-γ secretion and CD137, CD107a and granzyme B expression by RHAMM-specific T cells as compared to their RHAMM EP DC counterparts, despite the higher RHAMM protein levels found in the latter. This indicates that the basal levels of RHAMM expressed by mo-DCs are already sufficient to surpass the antigen threshold for T cell activation, which is in line with the observation that very few peptide/HLA complexes are required for the induction of complete T cell activation [27]. Presentation of TAAs that are expressed by *in vitro* generated DCs may at least in part explain why administration of TAA-“unloaded” DCs is able to inhibit tumor growth in mouse cancer models [28, 29]. On the other hand, concerns may rise about eliciting auto-immunity when targeting RHAMM, but so far none of the clinical vaccination studies with RHAMM peptide nor our own mo-DC trials noted significant toxicity [2, 20, 21].

To conclude, we established that mo-DCs currently used as autologous vaccines in cancer patients also express RHAMM and sufficiently present it to T cells in an HLA-dependent manner to allow full activation. Moreover, we identified RHAMM-specific T cells within the lymphocyte population infiltrating the vaccine sites of AML patients. This implies that clinical protocols with mo-DCs also vaccinate patients against RHAMM and not only against the TAA used to load the DCs.

## MATERIALS AND METHODS

### Experimental conditions and cell material

This study and reporting thereof is MIATA compliant (supplementary MIATA information) [30]. Experiments were approved by the Ethics Committee of the University of Antwerp, Antwerp, Belgium. Written informed consent was obtained for using leftover patient material from one of our clinical vaccine trials (clinicaltrials.gov - NCT00965224). Buffy coats were derived from anonymous healthy volunteers’ peripheral blood (with citrate phosphate dextrose anticoagulant) donated the day before and stored overnight at room temperature (Red Cross Blood Transfusion Center Antwerp, Edegem, Belgium). Peripheral blood mononuclear cells (PBMCs) were isolated from buffy coats by Ficoll density gradient separation (Ficoll-Paque PLUS, cat# 17-1440-03, GE Healthcare). T2 cells (cat# CRL-1992, American Type Culture Collection, Rockville, MD, USA), deficient for the transporter associated with antigen processing, were cultured in complete medium consisting of Iscove's Modified Dulbecco's medium (IMDM, cat# 21980-032, Thermo Fisher Scientific) with 10% fetal bovine serum (FBS; micro-organism pretested and qualified, cat# 10270-106, Thermo Fisher Scientific). The cytotoxic CD8^+^ T cell clone specific for the HLA-A*0201-restricted R3 epitope of RHAMM (amino acids 165-173: ILSLELMKL) was kindly provided by Dr. H. Bernhard (Technical University of Munich, Munich, Germany) [31]. In all experiments, cells were counted using an automated ABX Micros 60 cell counter (Horiba ABX) and (co-)cultures were maintained at 37°C. Cryopreserved cell samples were frozen down in FBS with 10% dimethyl sulfoxide (cat# D2650, Sigma-Aldrich) in a Nalgene Mr. Frosty™ Cryo 1 °C freezing container (cat# 5100-0001, ThermoFisher Scientific) at −80°C. All experiments were conducted in a biosafety level 1-certified laboratory that operates under exploratory research principles using established laboratory protocols and investigative research assays.

### RHAMM messenger RNA

The human *RHAMM* complementary (c)DNA sequence was modified for optimal codon usage and G/C content, flanked by the signal sequence and HLA class II-targeting sequence of DC-lysosome-associated membrane protein, [32] and subcloned into a pST1 plasmid vector, [33] putting it under the control of a T7 promoter and providing it with a poly(A)tail. Messenger (m)RNA transcripts were generated using a mMessage mMachine T7 *in vitro* transcription kit (cat# AM1344, Thermo Fisher Scientific) according to the manufacturer's protocol.

### DC generation

DCs from anonymous healthy volunteer donors were generated according to our AML clinical trial protocol (clinicaltrials.gov - NCT00834002, NCT00965224 and NCT01686334) with adaptations for research [34]. Briefly, CD14^+^ monocytes were positively selected from fresh PBMCs using CD14 MicroBeads (cat# 272-01, Miltenyi Biotec) according to the manufacturer's instructions and added to vented T175 culture flasks (cat# 353112, Corning) at a final concentration of 1.0-1.4 × 10^6^ cells/mL in 50-70 mL Roswell Park Memorial Institute (RPMI) 1640 medium (cat# 21875-034, Thermo Fisher Scientific) with 2.5% heat-inactivated human AB serum (micro-organism pretested and qualified, cat# 34005100, Thermo Fisher Scientific), 800 U/mL granulocyte-macrophage colony-stimulating factor (cat# CTK-221, Gentaur) and 20 ng/mL interleukin (IL)-4 (cat# PHC0041, Thermo Fisher Scientific). After 5 days, 20 ng/mL tumor necrosis factor-α (cat# CTK-223, Gentaur) and 2.5 μg/mL prostaglandin E2 (Prostin E2, Pfizer) were added to induce maturation. DCs were harvested 40-44 hours later and used as such (non EP DCs) or following electroporation without mRNA (mock EP DCs) or with 10 μg *RHAMM* mRNA (RHAMM EP DCs) in 200 μL Opti-MEM I reduced serum medium without phenol red (cat# 11058-021, Thermo Fisher Scientific). Immediately following electroporation, DCs were collected and washed in RPMI 1640 medium supplemented with 2.5% human AB serum and used in further experiments.

DCs from AML patients were thawed from cryopreserved aliquots and rested for two hours, before further use in experiments in adherence to our clinical trial protocol. Using good manufacturing practice-certified products, these DCs had been generated similarly as described above for non EP DCs (controls for trial immunomonitoring) or with the addition of KLH during maturation and electroporation with *WT1* mRNA following harvest (WT1 EP DCs; i.e., the clinical vaccine product) [2].

### RHAMM protein antibody staining

Four hours after electroporation (healthy volunteer donor samples) or two hours after thawing (patient samples), DCs were stained with LIVE/DEAD® Fixable Red Dead Cell Stain (cat# L23102, Thermo Fisher Scientific) prior to fixation and permeabilization for intracellular staining using the BD Cytofix/Cytoperm™ Fixation/Permeabilization Kit (cat# 554714, BD Biosciences) according to the manufacturer's instructions. Subsequently, samples were incubated with mouse anti-RHAMM monoclonal antibody (mAb; clone 2D6, cat# MO20030, Neuromics) or isotype control mouse IgG1 mAb (cat# 349040, BD Biosciences) followed by PE-labeled rat anti-mouse IgG1 mAb (clone X56, cat# 340270, BD Biosciences). RHAMM expression in viable (LIVE/DEAD^−^) cells was determined on a FACScan flow cytometer (BD Biosciences).

### *RHAMM* mRNA qPCR

Total RNA was prepared from 5 × 10^5^ monocytes or mo-DCs immediately after magnetic bead selection or electroporation, respectively, using the RNeasy Micro Kit (Qiagen). RNA concentration and purity were measured on a NanoDrop ND-1000 spectrophotometer. Approximately 500 ng of each RNA sample was used for synthesizing the cDNA with the iScript™ Advanced cDNA Synthesis Kit for RT-qPCR (Bio-Rad) which later served as template for a SYBR Green I-based qPCR on a LightCycler 480 II (Roche) using the LightCycler 480 SYBR Green I Master kit (Roche) and the following primers (Operon and Biolegio): RHAMM fw 5′-CAGGTCACCCAAAGGAGTCTCG-3′; RHAMM rv 5′-CCACTTGATCTGAAGCACAACTAA-3′; GAPDH fw 5′-TGCACCACCAACTGCTTAGC-3′; GAPDH rv 5′-GGCATGGACTGTGGTCATGAG-3′; YWHAZ fw 5′-ACTTTTGGTACATTGTGGCTTCAA-3′; YWHAZ rv 5′-CCGCCAGGACAAACCAGTAT-3′ [18]. All procedures were performed according to the respective manufacturer's instructions. RHAMM primers were designed to indifferentially amplify the 4 described native isoforms of RHAMM [9]. Results were analyzed using the ΔCt method and normalized to the mean of the expression of the GAPDH and YWHAZ housekeeping genes.

### RHAMM R3-specific cytotoxic CD8^+^ T cell clone activation

The RHAMM R3-specific T cell clone was thawed and cultured in triplicate with mock EP DCs, RHAMM EP DCs or WT1 EP DCs from HLA-A*0201^−^ or A*0201^+^ donors at a DC:T cell ratio of 4:1 in IMDM supplemented with 1% human AB serum in 96-well round bottom microplates (cat# 3799, Corning) or sterile capped 5 mL tubes (cat# 352054, Corning). Cultures of DCs or T cells alone and cultures of T cells with T2 cells ± 5 μg/mL of R3 or of R5 (RHAMM amino acids 274-282: SLEENIVIL) peptide (kind gift by Dr. J. Greiner, University of Ulm, Ulm, Germany) served as controls. After 24 hours, supernatants were collected and cryopreserved at −20°C for IFN-γ quantification or cells were analyzed by flow cytometry for CD137, CD107a and granzyme B expression.

### IFN-γ ELISA

IFN-γ secretion was quantified with a human IFN-γ ELISA kit (cat# 900-K27, Peprotech) according to the manufacturer's protocol. Standards and samples were measured in duplicate and triplicate, respectively, in a 96-well flat bottom microplate (cat# 439454, Nunc) on a Victor^3^ multilabel counter (Perkin Elmer).

### CD137, CD107a and granzyme B staining

After 24 hours culture, cell samples were stained with LIVE/DEAD® Fixable Aqua Dead Cell Stain (cat# L34957, Thermo Fisher Scientific), anti-CD3-PerCP-Cy5.5 (cat# 560835, BD Biosciences), anti-CD8-Pacific Blue (cat# MHCD0828, Thermo Fisher Scientific), anti-CD137-PE (cat# 555956, BD Biosciences) and anti-CD107a-FITC (cat# 555800, BD Biosciences) for 15 min at room temperature. Next, CD137 and CD107a expression by viable (LIVE/DEAD^−^) CD3^+^ CD8^+^ T cells was measured on a FACSAria II flow cytometer (BD Biosciences).

Granzyme B production was measured using a flow cytometry-based intracellular staining assay. After 1 hour of DC/T cell co-culture, 1 μL/mL protein transport inhibitor (GolgiPlug, cat# 555029, BD Biosciences) was added for the remaining 23 hours. After 24 hours, cells were stained with LIVE/DEAD® Fixable Aqua Dead Cell Stain (cat# L34957, Thermo Fisher Scientific), anti-CD3-PerCP-Cy5.5 (cat# 560835, BD Biosciences) and anti-CD8-Pacific Blue (cat# MHCD0828, Thermo Fisher Scientific) for 15 min at room temperature. Next, cells were fixed and permeabilized using the Foxp3/transcription factor staining buffer kit (cat# 00-5523-00, eBioscience) according to the manufacturer's instructions with minor adaptations. Cells were fixed by incubation with fix/perm working solution for 1 hour at 4°C, followed by two washing steps with permeabilization buffer. Permeabilized cells were stained with anti-granzyme B-FITC (cat# 560211, BD Biosciences) for 1 hour at 4°C. Samples were measured on a FACSAria II flow cytometer (BD Biosciences).

### RHAMM tetramer staining

Eight weeks after starting biweekly intradermal vaccination (i.e., after 4 vaccines) into the upper arms near the axilla, a DTH skin test was performed on the upper back with single intradermal injections of the vaccine or its components, including KLH alone. Two days later skin reactions were measured and skin biopsies were taken. DTH skin test-infiltrating lymphocytes (DILs) were collected and allowed to expand for 2-3 weeks in medium with 100 IU/mL of IL-2. After harvest, RHAMM-specific CD8^+^ T cells were quantified in DILs through flow cytometry following staining with R3 or R5 peptide-loaded (p)HLA-A*0201 tetramers (Fred Hutchinson Cancer Research Centre Seattle). For each condition, 1 × 10^6^ viable thawed DILs were washed and stained for 30 min at 4°C with allophycocyanin-labeled pHLA-A*0201 tetramers. Samples were then stained with LIVE/DEAD® Fixable Aqua Dead Cell Stain (cat# L34957, Thermo Fisher Scientific), anti-CD3-PerCP-Cy5.5 (cat# 560835, BD Biosciences), anti-CD14-FITC (cat# 345784 BD Biosciences), anti-CD19-FITC (cat# 345776, BD Biosciences) and anti-CD8-Pacific Blue (cat# MHCD0828, Thermo Fisher Scientific) for another 30 min at 4°C, followed by detection on a Cyflow ML flow cytometer (Partec). Dead (LIVE/DEAD^+^) cells and monocytes and B cells (FITC^+^) were excluded from analysis.

### Data analysis

Raw data were routinely checked for consistency and plausibility and can be provided upon motivated request. All samples were compared empirically to their respective negative and positive controls to determine reactivity and whether they fell within the ranges of the assay. Flow cytometry data were analyzed using FlowJo vX.0.6 (Treestar). GraphPad Prism 5 software was used for data comparison, statistical calculations and artwork. Statistical analysis was performed using one-way analysis of variance (ANOVA) with Bonferroni posthoc test or two-tailed unpaired *t* test or Mann Whitney *U* test. Differences were predefined to be considered as statistically significant when *P* < 0.05.
